# Neonatal Outcomes of Late-Preterm Birth Associated or Not with Intrauterine Growth Restriction

**DOI:** 10.1155/2010/231842

**Published:** 2010-03-22

**Authors:** Cristiane Ortigosa Rocha, Roberto Eduardo Bittar, Marcelo Zugaib

**Affiliations:** Department of Obstetrics, University of Sao Paulo, Av. dr Eneas de Carvalho Aguiar, 945, Cerqueira Cesar, Sao Paulo SP, cep 05412-002, Brazil

## Abstract

*Objective*. To compare neonatal morbidity and mortality between late-preterm intrauterine growth-restricted (IUGR) and appropriate-for-gestational-age (AGA) infants of the comparable gestational ages (GAs). *Methods*. We retrospectively analyzed neonatal morbidity and mortality of 50 singleton pregnancies involving fetuses with IUGR delivered between 34 and 36 6/7 weeks of GA due to maternal and/or fetal indication. The control group consisted of 36 singleton pregnancies with spontaneous preterm delivery at the same GA, in which the infant was AGA. Categorical data were compared between IUGR and AGA pregnancies by *X* ^2^ analysis and Fisher's exact test. Ordinal measures were compared using the Kruskal-Wallis test. *Results*. The length of stay of newborns in the nursery, as well as the need for and duration of hospitalization in the neonatal intensive care unit, was longer in the group with IUGR. Transient tachypnea of the newborn or apnea rates did not differ significantly between the IUGR and AGA groups. IUGR infants were found to be at a higher risk of intraventricular hemorrhage. No respiratory distress syndrome, pulmonary hemorrhage or bronchopulmonary dysplasia was observed in either group. The frequency of sepsis, thrombocytopenia and hyperbilirubinemia was similar in the two groups. Hypoglycemia was more frequent in the IUGR group. No neonatal death was observed. *Conclusion*. Our study showed that late-preterm IUGR infants present a significantly higher risk of neonatal complications when compared to late-preterm AGA infants.

## 1. Introduction

Prematurity is the leading cause of neonatal morbidity and mortality [1, 2]. Another important cause of perinatal morbidity and mortality is intrauterine growth restriction (IUGR), a condition in which the fetus is undernourished for gestational age [3]. Normally, IUGR is present in only a small percentage of deliveries, but an increased frequency has been observed among women who go into preterm labor followed by premature delivery [4]. 

Late-preterm birth is defined as birth between 34 weeks and 36 6/7 weeks of gestation [5]. During the past decade, the proportion of all U.S. births defined as late-preterm births has increased by 16% [6]. The overall rate of preterm births in the United States increased from 10.9% in 1990 to 12.8% in 2007, an increase of 16.6% [7]. This increase was mainly due to an increase in late-preterm births. In Brazil, approximately 88% of the 188,223 pre-term births recorded in 2005 occurred at an gestational age above 32 weeks [8].

The prevalence of IUGR is high in high-risk pregnancies. As a consequence, this condition is common among elective preterm deliveries and is therefore associated with late prematurity, with the observation of a recent increase in the incidence of these electively delivered late-preterm infants [4].

There are conflicting findings in the literature regarding outcomes of preterm infants with IUGR and appropriate-for-gestational-age (AGA) infants. Neonatal morbidity and mortality have been reported to be decreased [9–11], unchanged [12, 13] and increased [14–18] in IUGR infants compared to AGA infants.

The aim of this study was to compare neonatal morbidity and mortality between late-preterm IUGR and AGA infants of the same gestational age.

## 2. Methods

We retrospectively analyzed 86 singleton pregnancies, including 50 pregnancies involving infants with a birth weight of or below the 10th percentile (IUGR) delivered between 34 weeks and 36 6/7 weeks of gestation due to maternal and/or fetal indications. The control group consisted of 36 singleton pregnancies with spontaneous preterm delivery at the same gestational age, in which the birth weight ranged from the 11th to 89th percentile (AGA). The study was performed between 2005 and 2007. Birth weight percentiles were based on the standard growth curve of Alexander et al. [19]. 

Pregnancies complicated by diabetes (preexisting or gestational) and premature membrane rupture, pregnancies with fetal anomalies and pregnancies with unknown or conflicting dating criteria were excluded. 

Maternal characteristics included age, preexisting medical problems and pregnancy complications. Delivery characteristics included gestational age at delivery, birth weight, route of delivery, indication of elective birth, and Apgar scores. Neonatal data included death, transient tachypnea of the newborn (TTN), neonatal sepsis, intraventricular hemorrhage (IVH), hypoglycemia, jaundice, total number of days the infant was in the neonatal intensive care unit (NICU), and length of hospital stay. Gestational age at delivery was defined based on the mother's last menstrual period and was confirmed by early ultrasound examination. 

Preexisting medical problems included hypertensive disorders (chronic hypertension and pre-eclampsia), heart diseases, systemic lupus erythematosus and others (pulmonary disease, hepatitis, thrombophilia, anemia, etc.). Antepartum complications included oligohydramnios and fetal distress. Possible signs of fetal distress were a constant decrease in fetal heart rate variability, the occurrence of late or variable decelerations upon cardiotocography, or a high systolic/diastolic ratio in the umbilical artery. Amniotic fluid volume was estimated during the evaluation of the fetal biophysical profile. Neonatal acidosis was defined as an arterial umbilical cord pH less than 7.2 [20].

Diagnostic criteria for each neonatal problem are applied concurrently by neonatologists as follows: (1) TTN: clinical and radiographic features identified during the first hours of life, followed by characteristic resolution during the subsequent 24–48 hours; (2) neonatal sepsis: positive blood culture and clinical manifestations, or clinical manifestations, radiologic findings and laboratory indicators; (3) IVH: identified by serial cranial ultrasonography (all infants have head US); (4) hypoglycemia: blood glucose level below 40 mg/dL; (5) hyperbilirubinemia; (6) neonatal thrombocytopenia: platelet count less than 150.000/*μ*L (150 × 10^9^/L); (7) apnea of prematurity: prolonged respiratory pause (20 s or longer) accompanied by cyanosis, pallor or bradycardia.

The discharge criteria for preterm infants included weight > 2 kg and good suction upon breast-feeding accompanied by adequate weight gain.

Categorical data were compared between IUGR and AGA pregnancies by *X* ^2^ analysis and Fisher's exact test. Ordinal measures were compared using the Kruskal-Wallis test. IUGR was considered to be significantly related to outcome when *P* < .005.

## 3. Results

Of the 86 neonates included in the study, 50 belonged to the IUGR group and 36 to the AGA group. There was no significant difference in maternal age which ranged from 16 to 45 years (mean ± standard deviation: 25.1 ± 5.5 years) (*P* > .05). There was a predominance of white women (66.3%, *n* = 57), with no significant difference between groups. Among mothers of the IUGR group, 39 (78%) presented some underlying disease or obstetric complication in addition to IUGR, whereas 11 (22%) did not. Hypertensive syndromes were the most frequent condition and were observed in 24 (48%) women of the IUGR group. Heart disease was observed in 5 (10%) mothers of this group, systemic lupus erythematosus in 4 (8%), and other underlying diseases in 6 (12%) (pulmonary disease, hepatitis, thrombophilia, anemia, etc.) (Table 1). 

Gestational age at delivery ranged from 34 to 36.9 weeks (mean ± standard deviation: 35.5 ± 0.7, median: 35.6 weeks) and did not differ between groups. Preterm induction and preterm cesarean delivery were observed in 39 (78%) women of the IUGR group, whereas 11 (22%) patients went into spontaneous preterm labor. The indications for elective preterm delivery in the 39 patients of the IUGR group included oligohydramnios in 20 cases (51.3%), severe maternal disease in 8 (20.5%), presence of fetal maturity in 2 (5.1%), and abnormalities upon cardiotocography, fetal biophysical profile or umbilical artery Doppler in 9 (23.1%) (Table 1).

There was a significant difference in mean birth weight between the two groups (IUGR: 1810 g and AGA: 2695 g, *P* = .0001). The frequency of cesarean sections was 92% in the IUGR group and 25% in the AGA group (*P* < .0001). No difference in mean umbilical cord pH or the presence of neonatal acidosis was observed between groups. Only one newborn of the IUGR group and none of the AGA infants presented Apgar scores <7 at 5 minutes.

The length of stay of the newborn in the nursery, as well as the need for and duration of hospitalization in the NICU, differed significantly between the two groups (Table 2).

TTN or apnea rates did not differ significantly between IUGR and AGA infants. Late-preterm IUGR infants were found to be at a higher risk for IVH. There were only Grade 1 IVH in this sample. No respiratory distress syndrome, pulmonary hemorrhage or bronchopulmonary dysplasia was observed in either group. The frequency of sepsis or thrombocytopenia did not differ between groups. Hypoglycemia was more frequent in the IUGR group. The presence of hyperbilirubinemia was similar in the two groups (98% in the IUGR group versus 100% in the AGA group) (*P* = .52, Fisher's exact test) (Table 3). However, there was a difference in the number of days the newborn required phototherapy between the IUGR and AGA groups, which was higher in the former (Table 2).

None of the newborns died or developed retinopathy of prematurity or necrotizing enterocolitis during their stay in the nursery.

## 4. Discussion

Late-preterm births account for 70% of all preterm births [21]. Since the number of late-preterm births is increasing, it is important to understand the unique problems that this growing population of infants may experience. In this respect, a higher incidence of neonatal mortality has been reported for births that occur between 34 weeks and 36 weeks and 6 days [22] when compared to term newborns (>37 weeks). In addition, an increase in various neonatal complications such as hypoglycemia, respiratory distress syndrome (RDS), thermal instability, apnea of prematurity, hyperbilirubinemia, neonatal sepsis, prolonged stay in the nursery, and need for NICU treatment has been observed [22–28].

IUGR is a frequent complication in preterm infants and is the cause of most elective late-preterm deliveries. IUGR may also contribute to the increased morbidity and mortality observed among late-preterm infants. These infants are at risk for hypoglycemia, IVH, prolonged hospital stay and increased need for NICU treatment when compared to AGA infants, thus demonstrating the greater severity of these cases.

There is a lack of studies regarding the evolution of late-preterm infants with and without IUGR. Gilbert and Danielsen [29] reported a higher incidence of IVH and higher hospital costs for late-preterm IUGR infants when compared to AGA infants. In contrast to the present results, Sharma et al. [8] observed a lower incidence of RDS in late-preterm infants with IUGR; however, as observed in the present study, these infants required prolonged hospitalization in the nursery. Tyson et al. [15] and Piper et al. [30] compared the incidence of RDS between IUGR and AGA infants but we could not compare our results to theirs because these authors used a different definition of RDS. 

Although we did not observe more severe neonatal complications in the present cases (e.g., respiratory distress syndrome, bronchopulmonary dysplasia, pulmonary hemorrhage, necrotizing enterocolitis and neonatal death), hypoglycemia, neonatal sepsis, IVH, thrombocytopenia and hyperbilirubinemia were present in the late-preterm infants studied and were the cause of NICU treatment and prolonged stay in the nursery. The lack of observation of these more severe complications might be explained by the small sample studied since the frequency of these complications is rare in this gestational age group. Mean gestational age was 35.5 weeks in the two groups, a gestational age at which the incidence of respiratory distress syndrome is very low [31]. Thus, the number of newborns necessary to detect cases of this disease would have to be high. In the present series no cases of severe pulmonary complications such as bronchopulmonary dysplasia or pulmonary hemorrhage were observed, a finding that might be explained by the small sample size since these complications are also rare in this gestational age group [25]. The absence of neonatal death in the present sample is probably due to the low mortality of these newborns, which is approximately 7.7 per 1000 live births [21].

Despite the low rates of severe neonatal complications, these newborns are of marked importance for public health since they account for a large percentage of newborns hospitalized in NICUs and require large amounts of public resources during and after their stay in the nursery [32–34]. In addition, our study only analyzed neonatal morbidity immediately after delivery during the stay of the newborn in the nursery, but not the long-term consequences of late-preterm birth. The Institute of Medicine analyzed the late consequences of preterm birth in the United States and demonstrated marked human and economic impacts during childhood resulting from preterm birth [35]. 

As also reported in the study of Gilbert and Danielsen [29], the frequency of IVH differed significantly between the two groups and was more common in IUGR infants, a finding suggesting that IUGR is indeed a risk factor for IVH in late-preterm infants. Laptook and Jackson [36] have demonstrated an elevated incidence of hypoglycemia in late-preterm infants as a result of deficient neoglycogenesis, hepatic glycogenolysis and lipolysis and of hormonal irregularities. Neonatal sepsis was rare in the present sample and was similar in the two groups (4% versus 0). These findings agree with Arnon et al. [24] who observed an incidence of 5% of neonatal sepsis in neonates born at 34 weeks of gestation and no case among those born at 36 weeks. The exclusion of cases with premature membrane rupture may have contributed to this result [24]. Neonatal jaundice was very common in both groups (98% versus 100%). Furthermore, IUGR infants required phototherapy for a longer period of time than AGA infants, that is, jaundice was more severe in this group.

Late-preterm birth poses various risks to the newborn and the obstetrician should always weigh the risks and benefits in each case to decide whether to interrupt pregnancy between 34 weeks and 36 weeks and 6 days of gestation. We believe that the technological advances in obstetrics that have occurred over the last few years permit a better control of high-risk pregnancies. Thus, the priority of the obstetrician is to strive for delivery as close to term as possible.

## 5. Conclusion

In conclusion, our study showed that late-preterm IUGR infants present a significantly higher risk of neonatal complications and a significantly longer NCIU and hospital stay when compared to late-preterm AGA infants. Thus, evaluation of the birth weight percentile for gestational age may provide a more realistic expectation of outcome among late-preterm infants.

## Figures and Tables

**Table 1:** Maternal characteristics and indications for elective preterm delivery in the IUGR group (*n* = 50).

Underlying disease/		Indications for	
obstetric complications		elective resolution	
Systemic	4 (8%)	Oligohydramnios	20 (51.3%)
Lupus			
Erythematosus			

Heart diseases	5 (10%)	Severe maternal	8 (20.5%)
		Disease	

Hypertensive	24 (48%)	Cardiotocographic	6 (15.4%)
Disorders		Abnormalities	

Others	6 (12%)	FBP or	3 (7.7%)
		Doppler alterations	
		Fetal maturity	2 (5.1)

IUGR: intrauterine growth restriction; FBP: fetal biophysical profile. Data are reported as number of cases and percentage.

**Table 2:** Comparison of neonatal outcomes between the IUGR and AGA groups.

Outcome		IUGR group	AGA group	*P*
Length of stay	Mean	16.36	4.58	
(days)	Median	16.5	3	.0001
	SD	10.77	2.18	

Length of	Mean	5.92	1.28	
NICU stay	Median	2.5	0	<.0001
(days)	SD	7.71	2.28	

Phototherapy	Mean	5.78	3.19	
(days)	Median	5	3	**.005**
	SD	3.71	2.11	

IUGR: intrauterine growth restriction; AGA: appropriate-for-gestational age; NICU: neonatal intensive care unit; SD: standard deviation.

**Table 3:** Comparison of neonatal complications between the IUGR and AGA groups.

Neonatal	IUGR group	AGA group	*P*
complications			
TTN	27 (54%)	16 (44.4%)	>.05
Apnea of	3 (6%)	0	>.05
prematurity			

Intraventricular	6 (12%)	0	.037
hemorrhage			

Neonatal	5 (10%)	0	>.05
thrombocytopenia			

Neonatal sepsis	2 (4%)	0	>.05

Hypoglycemia	12 (24%)	2 (6%)	.047

Hyperbilirubinemia	49 (98%)	36 (100%)	.52

Data are reported as number of cases and percentage. IUGR: intrauterine growth restriction; AGA: appropriate-for-gestational age; TTN: transient tachypnea of the newborn.
